# Early Osseous Proliferation in Spiraled Healing Chambers Resulted After the Insertion of Titanium Implants in Cortical Bone of a Rabbit

**DOI:** 10.3390/medicina62010072

**Published:** 2025-12-29

**Authors:** Cristian Adrian Ratiu, Danut Dejeu, Camelia Anca Croitoru, Adrian Todor, Ioana Adela Ratiu, Ruxandra Elena Luca, Corina Moisa, Viorel Miclaus, Vasile Rus

**Affiliations:** 1 Faculty of Medicine and Pharmacy, University of Oradea, 1st December Square 10, 410073 Oradea, Romania; cristian.ratiu@didactic.uoradea.ro (C.A.R.); ddejeu@uoradea.ro (D.D.); camelia.croitoru@didactic.uoradea.ro (C.A.C.); 2 Discipline of Oral Implantology, Dentistry Department, Faculty of Medicine and Pharmacy, University of Oradea, 1st December Square 10, 410073 Oradea, Romania; 3 Surgery Department, Emergency Clinical Hospital Bihor County, 12 Corneliu Coposu Street, 410469 Oradea, Romania; 4 Faculty of Medicine and Pharmacy, University of Medicine and Pharmacy “Iuliu Hatieganu” Cluj-Napoca, 8 Victor Babeș Ave., 400347 Cluj-Napoca, Romania; atodor81@gmail.com; 5 Nephrology Department, Emergency Clinical Hospital Bihor County, 12 Corneliu Coposu Street, 410469 Oradea, Romania; 6 University Clinic of Oral Rehabilitation and Dental Emergencies, Faculty of Dentistry, “Victor Babes” University of Medicine and Pharmacy, Eftimie Murgu Square No. 2, 300041 Timișoara, Romania; 7 Interdisciplinary Research Center for Dental Medical Research, Lasers and Innovative Technologies, Revolutiei 1989 Avenue No. 9, 300070 Timișoara, Romania; 8 Department of Pharmacy, Faculty of Medicine and Pharmacy, University of Oradea, 1st December Square 10, 410073 Oradea, Romania; cmoisa@uoradea.ro; 9 Faculty of Veterinary Medicine, University of Agricultural Sciences and Veterinary Medicine in Cluj-Napoca, Calea Mănăştur 3-5, 400372 Cluj-Napoca, Romania; vmiclaus@usamvcluj.ro (V.M.); vasile.rus@usamvcluj.ro (V.R.)

**Keywords:** healing chamber, implant, osseointegration

## Abstract

*Background and Objectives*: The insertion of endosseous implants requires the alveolar bone to be drilled, which produces alterations of the osseous neoalveolus approximately 1 mm deep, an area that will later be subjected to osseous renewal. The healing of the bone around the inserted implant is complex and depends on numerous factors, amongst which the size of the insertion orifice relative to the diameter of the implant, the design, and the pace and depth of the threads play an essential part. Therefore, the aim of this paper is to investigate from a histologic point of view the osseointegration of the implants inserted in a rabbit cortical bone by creating a 150 µm high healing chamber. *Materials and Methods*: 5 mm-long and 2 mm-wide titan implants were inserted into the femur of 15 12-month-old rabbits by using a drill with a 1.8 mm diameter, obtaining a spiralled healing chamber 150 µm high. The animals were euthanized after 7, 14, and 28 days according to effective legal and ethical protocols. The bone around the implants was severed 5 µm thick. After coloring with the Tricrom Goldner method, the sections that intercepted most centrally the intervention area were examined and photographed with an Olympus microscope. *Results*: The histologic result showed osseous healing within the healing chamber in the third to the endosteum of the implant after 7 days from the insertion. After 14 days, the osseous healing spread to 2/3 of the healing chamber. After 28 days, the whole healing chamber was occupied by bone. *Conclusions*: The healing chamber favored proper conditions for osseous healing, which began at the level of the endosteum. This statement is based on the histologic findings of bone formation after 7 days only in the third of the endosteum of the healing chamber. A 150 µm height of the healing chamber obtained in the rabbit cortical bone does not pose a risk of connective tissue proliferation.

## 1. Introduction

During their whole life, humans and animals may suffer injuries of different intensity or extent, from minor skin cuts to major injuries like fractures or major organ lesions. When the body faces such major traumatic events, it turns to certain mechanisms that, most of the time, manage to heal the damaged tissues. These healing mechanisms are not equally efficient in all animals, with some of the animals being able to regenerate whole parts of the body after an injury. The human, being a more evolved mammal, does not have such mechanisms, so that the recovery means are mostly those of repairing and, only in some cases, of regeneration of some components [1]. Regeneration is a type of healing in which the newly formed tissues manage to restore parts of the damaged tissue up to its normal state or very close to it. Restoration is a way of healing in which seriously damaged tissues are replaced with connective tissue, a phenomenon known as scarring. The two phenomena are not always totally separate, so that the greatest part of tissue reconstruction after lesions is achieved both by regeneration and by restoration. Note that the regenerated tissues regain the function they had before the injury. The tissues with mixed recovery will more or less regain their function, but those repaired with connective tissue will never regain their previous function [1].

The insertion of an implant in the bone is made by means of a surgical intervention, which comes with injuries at the site of the intervention. As a result, certain modifications at the level of the wall of the osseous orifice occur, respectively, tissular damage and the rupture of the blood vessels surrounding the site. Any modification that occurs as a consequence of surgical trauma can be considered normal only if 1 mm deep from the osseous wall surface of the experimental defect [2].

As the body is able to repair damaged structures, the area around the implant will immediately start healing. This process implies a special complexity, comprising a multitude of events, each of which having a certain share of taking part in the healing of the peri-implant tissues. The complexity of the healing process is made up of the multitude of factors that more or less influence the successive events during the different phases of the peri-implant healing. Among the factors that significantly influence peri-implant tissue healing stands out the type of bone that is being synthetized, whether cortical or trabecular, and the density of it. The primary stability of the implant directly depends on the quality of the bone in which the implant is inserted, which is decisive in the adequate progress of the reparatory processes. The severity and extent of the peri-implant tissular damage, and also the ratio of the insertion orifice diameter to the diameter of the implant, are paramount [3,4,5,6]. This ratio is of much importance, as it has to be adapted in such a manner so that it does not exert too much pressure on the bony wall, but also so that it offers primary stability, depending on the type of bone in which it is inserted. If the space is adequate, the best conditions for the early proliferation of the peri-implant tissues are created [3,7].

The implant may be threaded or non-threaded, but nowadays, the threaded implants are preferred as the shearing forces on the bone–implant interface offer a superior primary stability [8,9]. The advantages of the threaded implants are given by the fact that the threads enhance the primary stability of the implant and offer a better distribution of the stress loads [10].

Concerning the design of the thread, it can be square-shaped, V-shaped, buttress, or reverse buttress [11]. The functional surface, which determines the distribution of the bio-mechanical charges on the implants, is in direct relationship with the depth, the thickness, the end, and the helical angle of the thread [12]. The functional surface area per unit length of the implant may vary depending on the geometric characteristics of the thread, including pitch, profile, and depth. The pace of the thread represents the number of threads per unit of length in the same axial plane and on the same part of the axis of the implant body. The smaller the pace, the higher the number of threads in the body of the implant. Thus, the contact surface with the bone is increased. The type of bone in which the implant is placed must also be considered. In dense bone, a lower number of threads is preferable, whereas in low-density bone, a higher number of threads is recommended [9].

The shape of the thread represents a very important characteristic, as it may change the direction of the occlusal charge of the prosthetic in different directions in the bone. From the point of view of the axial charges, a triangular thread is comparable with a buttress thread at an approximately 30-degree angle. Some specialists assert that a square design of the thread reduces the component of the shearing force. The implant introduced by Bränemark in 1965 had triangular threads that were to be inserted with a threaded osteotomy [13]. This initial design was modified over the years to allow for a simpler and more efficient insertion, as well as a better distribution of the charges [9].

The depth of the thread is given by the distance between the smallest and the biggest diameter of the thread. In most types of implants, the depth of the thread is uniform on the entire length of the implant, but there are cases when this can vary to offer a bigger functional surface in the regions with the most intense loads, like the alveolar crests. A very important aspect is related to the proportions set between the surface of the implant and the surface of the drilled orifice for the insertion of the implant. In many cases, the size of the last drill used for the osteotomy corresponds to the diameter of the body of the implant. This way, the deeper the threads, the bigger the insertion of the implant (threads) into the osseous tissue is.

It is often recommended to use a drill with a slightly smaller diameter than that of the implant, allowing the implant to be inserted with a controlled degree of torque. We have to point out that the more the drilling place is undersized, the higher the insertion torque. Specialists remark that high levels of torque can provoke a powerful compression of the osseous tissue, which can lead to an osseous remodeling expanded in time [14]. In studies led on animals, it was noticed that the drilling of an orifice with a diameter close to the diameter of the implant favors a superior formation of new bone. In this case, a free space is created between the osseous wall and the bottom of the channel between the threads of the implant, a space that they named the healing chamber [9,15]. The presence of the healing chamber is beneficial for the osseointegration process, as it facilitates the proliferation of new bone on the interface [16]. The healing chamber is obtained by drilling the insertion orifice with a drill that has a bigger diameter than the central diameter of the implant (axis), but smaller than the total diameter of the implant (together with the threads). The empty space in the healing chamber facilitates rapid clotting and the employment of cells that take part in the rapid formation of bone [17,18]. Yet, we must take into account that this healing chamber reduces to some extent the contact surface between the implant and the insertion orifice wall, a reason for which this method of inserting is beneficial for high-density bones, but must be used cautiously in low-density bones [19]. The sizes of the healing chambers must fit certain limits. Some researchers argue that the maximum size of the healing chambers would be 150 μm because, above this limit, we run the risk of connective tissue being proliferated instead of bone [20,21].

The aim of this study is to follow the dynamic of the osseointegration process in a rabbit cortical bone by using 150 μm-high healing chambers to see if the risk of connective tissue proliferation occurs.

## 2. Materials and Methods

In order to perform this study, we received the agreement of the Bioethics Commission of the Agricultural Sciences and Veterinary Medicine University of Cluj-Napoca, registered with no. 384/20 August 2023. The requirements regarding the protection of animals for experiments were observed in conformity with the protocol of Amsterdam D 86/609/CEE and GO 37/2002.

### 2.1. Surgical Procedure

As biological material, we used 15 12-month mixed-race male rabbits kept in bio base conditions, respectively, a medium temperature of 22 °C, a day/night 12/12 ratio, standardized food (dry), and water at will.

**Figure 1:**
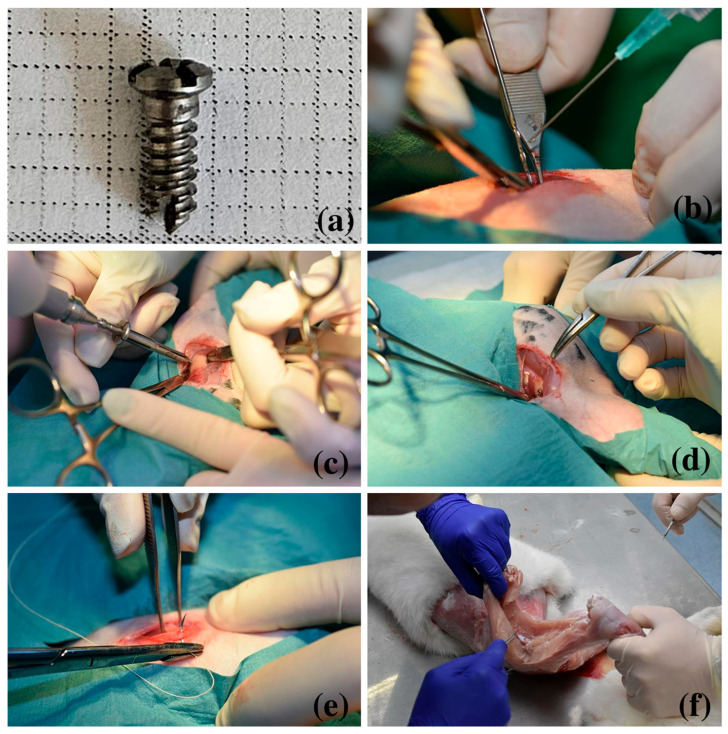
Material and Methods: Sequential steps of the implant placement procedure. (**a**) The screwed implant—5 mm long, 2 mm thick; (**b**) performing the insertion orifice with a 1.8 mm drill; (**c**) the insertion of the implant in the created orifice; (**d**) the screw implanted in the femoral bone diaphysis; (**e**) closing the surgical incision; (**f**) the stratigraphic dissection of the muscles to mark out the insertion place of the implant.

As testing materials, we used screwed implants 5 mm long and 2 mm in diameter (Figure 1a) made of titanium by Biomicron Transylvania, Cluj-Napoca, Romania. We chose these sizes to meet the recommendations of the field literature, where it is stated that the maximum size accepted for endosseous implants for rabbits is 6 mm long and 2 mm wide [22]. Implants with a bigger diameter may be used in larger species, including humans, but in rabbits, the risk of fracture increases together with the increase in diameter and could also overstress the animal [23].

The preparation of the animals for the surgical intervention began with the shaving of the area, followed by anesthesia with 5 mg/kg Xylazine and 35 mg/kg Ketamine. To maintain the anesthesia, we used a respirator with Isoflurane. During the intervention, fluid therapy was ensured by connecting the animal to a fluid therapy device. The intervention area was disinfected with iodine. Then, we made an incision through the skin and the soft tissues under the skin to the surface of the femoral bone. The insertion orifice was drilled with a 1.8 mm diameter drill (Figure 1b). The screws were inserted by hand by self-tapping with a special screwdriver (Figure 1c). A certain length was chosen for the screw so that it penetrates the periosteum, the osseous wall, the endosteum, and the marrow in the medullary channel. We used two implants per animal, one in the right leg and the other in the left leg. The insertion of a 2 mm diameter screw in an orifice of 1.8 mm diameter made the thread of the screw penetrate the bone 0.10 mm deep, thus ensuring the primary stability necessary for the osseointegration process. Also, the profound portion of the thread, 0.15 mm high, was left free so that a threaded gutter 150 μm high spread on the whole thickness of the osseous wall from the periosteum to the endosteum. This space was named the healing chamber. After the insertion of the screw (Figure 1d), we closed the surgical wound (Figure 1e). Post-op, we used subcutaneous Enrofloxacin 20 mg/kg for 5 days and Meloxicam 1 mg/kg for 3 days.

Post-op, we carried out daily clinical exams until the end of the experimental period. Moving to food and water bowls was difficult during the first three days due to pronounced limping, but the animals did not refuse food or water. After three days, the limping continuously decreased, and the animals became more and more active. Moreover, during the first 24 h, the intervention area was obviously tumid, but it soon ceased.

The animals were divided into three groups of 5, depending on the moment when the osseous proliferation was observed in the healing chambers. For the first group, the experiment lasted for 7 days, for the second group, 14 days, and for the third group, 28 days.

### 2.2. Histological Analysis

At the end of the experiment, the animals were euthanized with sodium phenobarbital. A stratigraphic dissection was performed to emphasize the place in the femur where the implant was inserted (Figure 1f), and their femur was harvested, which was split 5 mm proximally and distally from the implant. The pieces with the implants were introduced in a 10% neutral buffered formalin solution for fixation. The fixation of the samples took 7 days, during which the fixator solution was changed three times. At the end of the fixation period, the harvested samples were decalcified with 10% trichloracetic acid, dehydrated with ethylic alcohol, clarified with n-Butanol, and paraffin-embedded. Five μm-thick serial sections were performed, from which those that intercepted the intervention area most centrally were selected. The histological slides were stained using Goldner’s trichrome method. The examination of the histologic slides was conducted by two experienced specialists using an Olympus BX41 microscope (Evident Scientific, Tokyo, Japan). The images were taken with a digital camera, an Olympus E-330 (Olympus Corporation, Shinjuku, Japan).

## 3. Results

**Figure 2:**
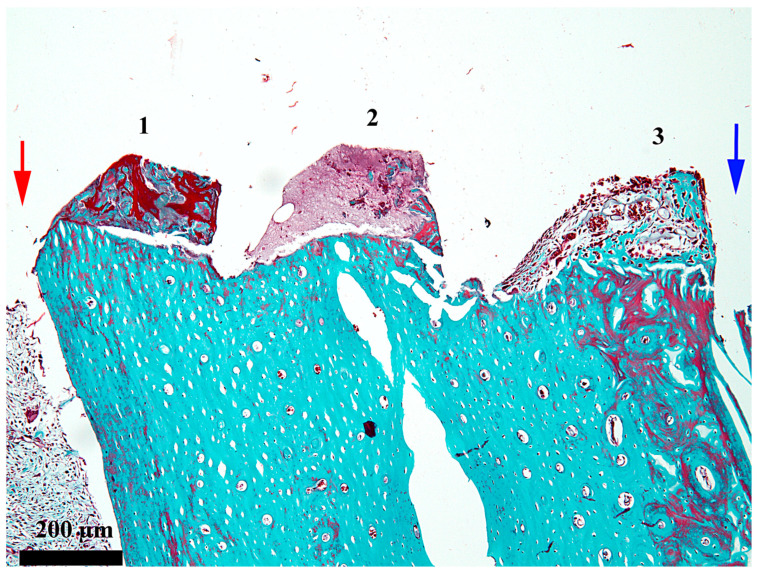
General aspect of the healing chamber after 7 days on the bone–implant interface; red arrow—periosteal area; blue arrow—endosteal area; 1—the external third of the healing chamber; 2—the middle third of the healing chamber; 3—the internal third of the healing chamber; Goldner’s trichrome stain.

**Figure 3:**
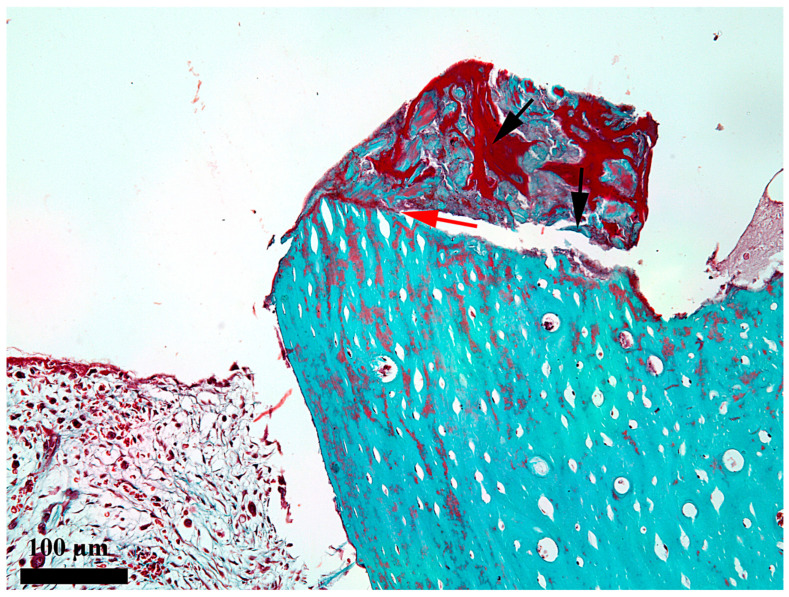
Microscopic detail of the content at 7 days after the insertion in the external third of the healing chamber; black arrow—bone detritus; red arrow—bone-healing chamber interface; Goldner’s trichrome stain.

**Figure 4:**
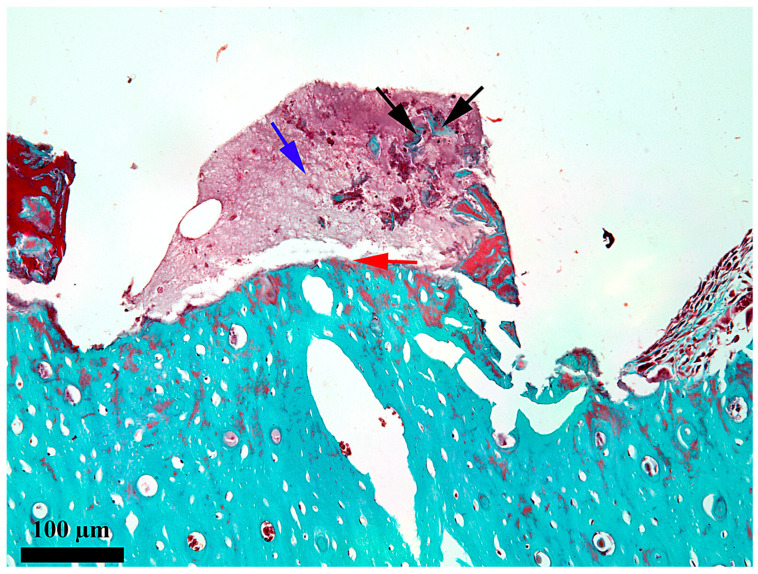
Microscopic detail of the content at 7 days after the insertion in the middle third of the healing chamber; black arrow—bone detritus; red arrow—bone-healing chamber interface; blue arrow—fibrin–leukocytic network; Goldner’s trichrome stain.

**Figure 5:**
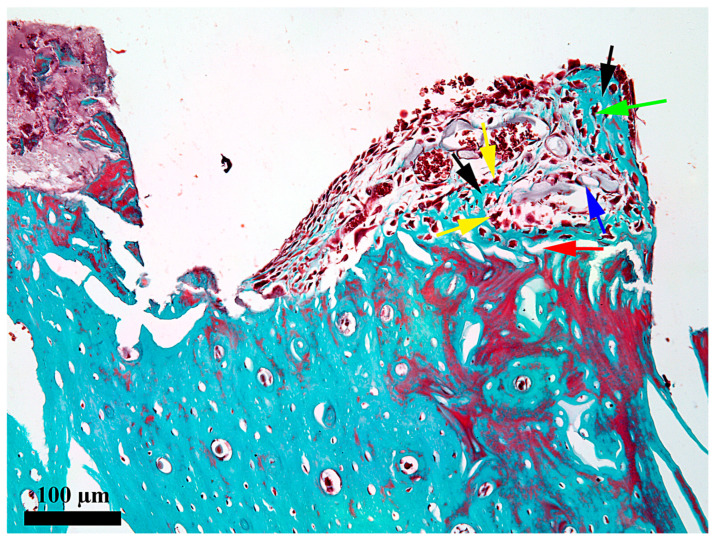
Microscopic detail of the content at 7 days after the insertion in the internal third of the healing chamber; red arrow—bone-healing chamber interface; black arrow—thin osseous trabeculae; yellow arrow—osteoblasts; green arrow—osteocyte; blue arrow—blood vessel in the areola; Goldner’s trichrome stain.

At 7 days after the insertion of the implant, the healing chamber seems occupied along its entire length, but the content differs according to its position related to the periosteum and endosteum, respectively (Figure 2). In the third, towards the periosteum (Figure 3), the healing chamber has a relatively dense content formed by a blood clot containing polymorph osseous particles, which was created here during the drilling and self-tapping necessary for the insertion of the implant. In the central part (Figure 4), the healing chamber is filled with a fibrin–leukocytic network that contains small osseous fragments and significantly fewer than those from the third towards the periosteum. In the third towards the endosteum (Figure 5), the situation differs a lot compared to the one already described, meaning we can notice here osseous-repairing processes in full development, even if these are in an incipient phase. The osseous formations present here are very young and delicate and tend to organize themselves as areolar bone surrounding the blood vessels, very well represented here after 7 days. In other words, in this area of the healing chamber, we can notice osseous repairing processes which, by their aspect, seem to have been started 3–4 days before day 7.

**Figure 6:**
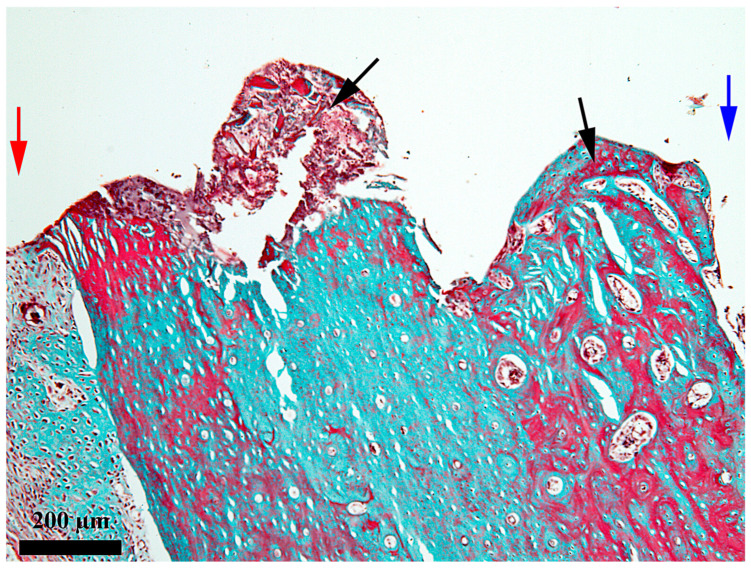
General aspect of the healing chamber on the bone-implant interface after 14 days; red arrow—periosteal area; blue arrow—endosteal area; black arrows—the content present in the healing chamber; Goldner’s trichrome stain.

**Figure 7:**
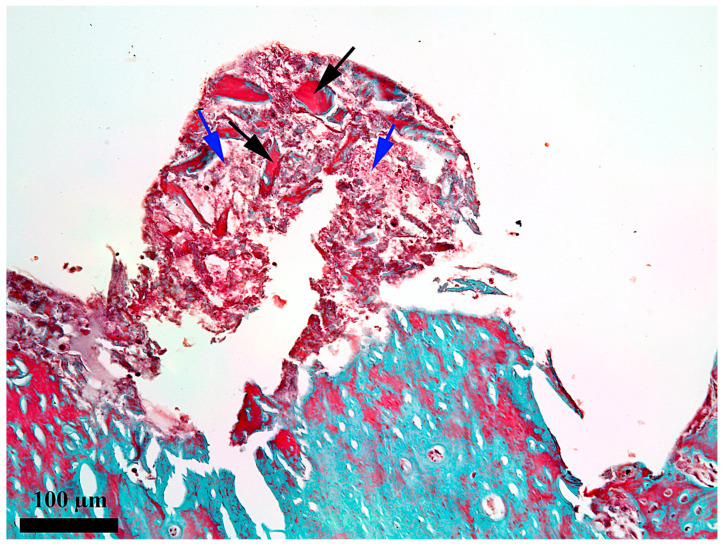
Microscopic detail of the content at 14 days after the insertion of the implant in the external third of the healing chamber; black arrow—bone detritus; blue arrow—fibrin–leukocytic network; Goldner’s trichrome stain.

**Figure 8:**
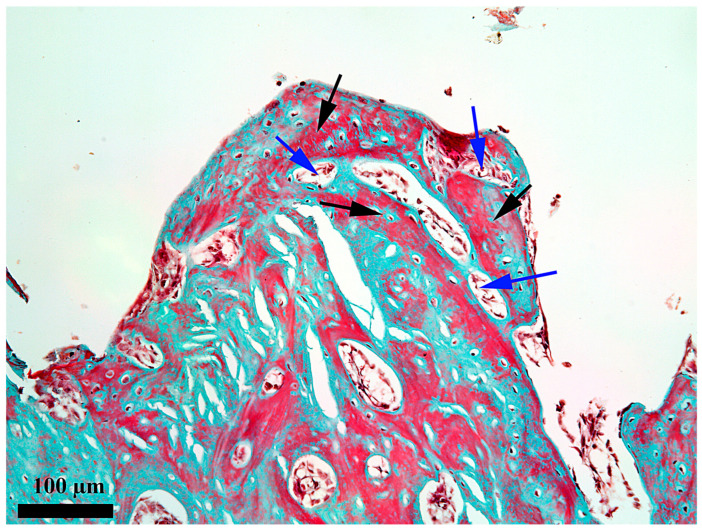
Microscopic detail of the content at 14 days after the insertion of the implant in the internal third of the healing chamber; black arrow—thick osseous trabeculae; blue arrow—small areolae; Goldner’s trichrome stain.

At 14 days after the implant insertion (Figure 6), the repairing processes from the healing chamber are obviously more advanced than on day 7, but still, the newly proliferated bone does not yet occupy the entire length of the healing chamber. If, on day 7, the newly proliferated bone partially fills the third towards the endosteum of the healing chamber, on day 14, the newly proliferated bone fills 2/3 of the length of the chamber, and only the third from the periosteum does not yet contain new proliferated bone, but a network of fibrin and bone detritus (Figure 7). This demonstrates a good evolution of the repair processes and the fact that their onset occurred in the third from the endosteum, from where it gradually extended into the middle third of the healing chamber. The bone present in the healing chamber in the endosteal (Figure 8) and middle thirds is woven bone with an areolar appearance.

**Figure 9:**
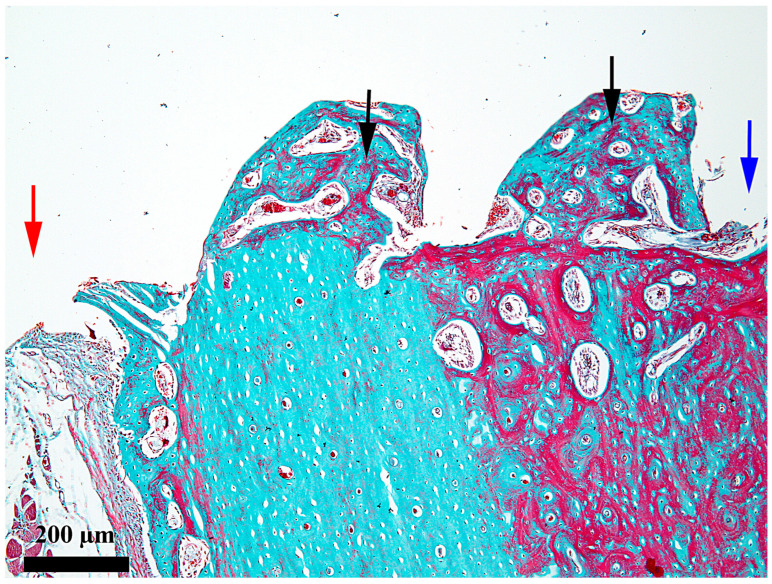
General aspect of the healing chamber on the bone–implant interface after 28 days; red arrow—periosteal area; blue arrow—endosteal area; black arrows—the content present in the healing chamber; Goldner’s trichrome stain.

**Figure 10:**
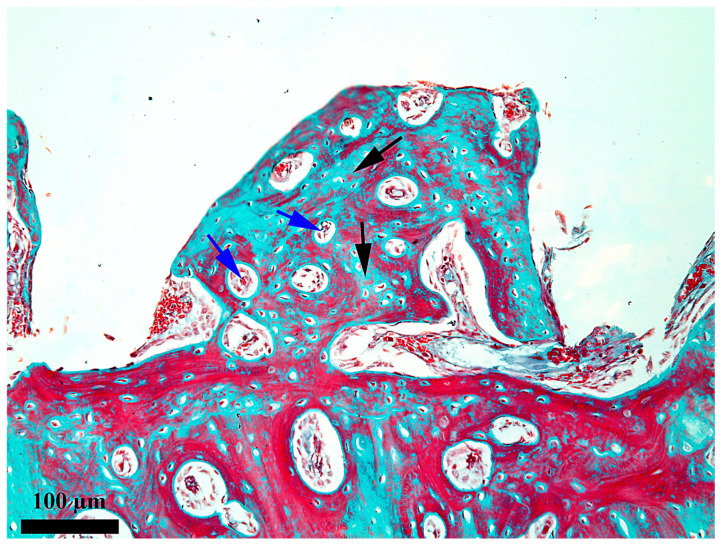
Microscopic detail of the content 28 days after the insertion in the internal third of the healing chamber; black arrow—thick osseous trabeculae; blue arrow—small areolae; Goldner’s trichrome stain.

**Figure 11:**
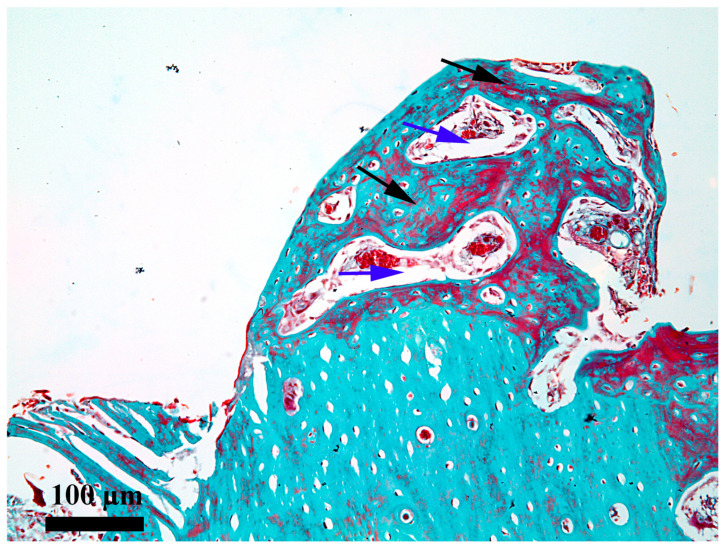
Microscopic detail of the content 28 days after the insertion in the external third of the healing chamber; black arrow—thick osseous trabeculae; blue arrow—large areolae; Goldner’s trichrome stain.

On day 28, the newly proliferated bone is present on the entire length of the healing chamber (Figure 9) from the periosteum to the endosteum. This bone fully occupies the healing chamber and has the aspect of an areolar bone, with some differences depending on the area. The small differences consist in the fact that the bone from the endosteum third (Figure 10) has the smallest areolae and that from the periosteum third (Figure 11) has the biggest, which demonstrates that it is not exactly in the same evolution stage along the whole length of the healing chamber. It is noted that, although this bone is mostly woven bone, there are processes of remodeling toward the lamellar bone, as demonstrated by the existence of outlines of bone lamellae that tend to be arranged somewhat circularly around the areolas. It should be noted, however, that the remodeling processes are in their early stages, occupying limited areas for now.

## 4. Discussion

The success of the osseointegration greatly depends on the stability of the implant, which is classified as primary or secondary. The stability of the implant is influenced by many factors, like the design of the implant, the topography of the surface of the implant, the quality of the bone, and the factors related to the patient. The primary stability is also called mechanical because it represents an interlock of the implant in the bone at the moment of the insertion of the implant. The secondary stability is also called biological because it is obtained by a series of events related to the osseous proliferation and remodeling. In this respect, the primary stability is related to the old bone, and the secondary stability is related to the newly proliferated bone [24].

The primary stability is ensured by the mechanical anchorage of the implant in the osseous wall, with the mechanical interlock between the threads of the implant and the bone they are penetrating. The mechanical interlock is, at this moment, the only interaction between the implant and the bone without any biological interaction. Based on these aspects, some specialists assert that the primary stability is only preparing the osseointegration and should not be considered as part of osseointegration [25].

The implant should be anchored so that its primary stability is ensured, but, at the same time, between the surface of the implant and the osseous wall of the defect in which the insertion is made, there needs to be a space in which the newly proliferated tissues can be laid. The types of tissues proliferated before the implant depend on the size of this space. The specialists came to the conclusion that these spaces should be a few μm to allow for the proliferation of small capillaries that are tens of μm to host whole cells [26,27]. For the formation of lamellar bone and especially of secondary osteons, these spaces have to be significantly larger. In this regard, some specialists came to the conclusion that spaces of 50 μm allow the formation of plexiform bone [28]. The lamellar bones need spaces over 100 μm, and for the formation of secondary osteons, the spaces have to be over 140 μm [29]. As we can notice, a larger space favors the proliferation of bone structures with higher complexity. The specialists are particular about the fact that this space should not exceed 150 μm, as the risk of the space being filled with connective tissue may occur. Such a situation is not desirable as peri-implant connective tissue proliferation compromises the osseointegration of the screw into the bone. A too-small remaining space is onerous, as such a space does not advantage the contact osteogenesis [2].

For an implant to be considered stable, the movements between it and the bone wall must be limited so that they allow a successful growth of tissue around the implant and the development of the angiogenesis and osteogenesis processes. The process of peri-implant tissue healing is somewhat similar to the healing of a bone fracture. In case of fractures, the instability between the bone ends determines the cartilage formation. If the instability exceeds a certain limit, pseudoarthrosis occurs [30]. Movement over a certain limit is harmful in the case of implants, too, as it can determine the growth of fibrous connective tissue to the detriment of bone growth. Some specialists assert that a micro movement higher than 150 μm can lead to fibrous tissue proliferation on the surface of the implant instead of bone [20].

The final aim of the peri-implant tissue repairing processes is the reconstruction, both quantity- and quality-wise, of the affected structures at a level as close as possible to the state before the trauma. In some specialists’ opinion, the only tissue in the body that can make a reconstruction very close to the state before the trauma is the bone tissue [31].

Our choice of implant thread insertion ensures the primary stability necessary for osseointegration, eliminating the risk of excessive tension. However, it does not guarantee intimate contact between the entire threaded surface and the bone wall. The intimate contact required for primary stability is achieved by the thread tips, which penetrate approximately 100 μm into the bone wall of the experimental defect. As the threads are 250 μm high, more than half of their height will not penetrate the bone, thus creating a space that is not filled by bone between the threads with the shape of a fillet from the periosteum to the endosteum. This fillet is 150 μm high and is called the healing chamber. Immediately after the insertion of the implant, the healing chamber is filled with blood, which contains heteromorphic bone fragments resulting from the drilling and self-tapping. In a short while, a clot incorporating bone fragments is organized in the healing chamber.

The surgery needed for the insertion of the implant is accompanied by trauma at the bone tissue level and the surrounding soft tissues. By tearing the blood vessels, the intervention area will be flooded with blood. In this context, the first tissue the implant comes in contact with when inserted in the bone is the blood, and this contact is immediately followed by a series of biological events, like protein bedding, coagulation, inflammation, and tissue formation [30]. The serum proteins brought by blood, like fibrinogen, albumin, and fibronectin, begin to adhere to the titanium surface in a very short period, like seconds or minutes [32,33]. The von Willebrand factor and the IgG are also absorbed on the surface of the implant, working towards platelet activation, coagulation, and inflammation [30]. The proteins are bedded in a single layer and prepare the underlying tissue for the joining and proliferation of the cells. This process begins during the first 24 h [34]. The platelets and the mesenchymal cells will interact with the proteins in this layer. The number of proteins in the blood exceeds 200, but not all of them bind to the surface of the implant at a marked level. The composition of the protein monolayer is mainly determined by the characteristics of the surface of the implant [30].

The platelets become involved to stop the bleeding. Being exposed to collagen or other proteins from the wounded tissue, they aggregate and close the ruptured blood vessel [35]. The contact of the platelets with the surface of a material determines their activation, followed by cytokine release, which activates other cells on the surface of the implant.

The platelets release thromboxane, which initiates the platelet aggregation and other substances, like Platelet-Derived Growth Factors (PDGF) and the Transforming Growth Factor-beta (TGF-β), which stimulate the division of the fibroblasts and vasoactive factors like histamine and serotonin. Both TGF-β and PDGF are chemotactic factors for neutrophils, fibroblasts, smooth muscle cells, and osteogenic cells [36]. Following the platelet degranulation, the metabolites of arachidonic acid are also released, which bring vasoconstriction.

The III and VII factors from the blood that moved into the area activate the X factor, which, together with the V factor, transform the prothrombin into thrombin. Then, the thrombin breaks down the fibrin peptides from the fibrinogen, resulting in the fibrin of the clot [37]. By spontaneous reticulation, the fibrin monomers organize in a fibrin network, taking part in the organizing coagulation. The resulting blood clot fills the space of the wound and sticks to the surface of the implant, resulting in a temporary matrix. This temporary matrix plays an essential role in the development of the further processes of bone healing on the surface of the implant [35].

The building up of a stable clot gives both the mechanical and biological components necessary for the osseointegration [30].

By filling the spaces in which the bone does not come into close contact with the screw, the clot is an excellent autologous augmentation material [38], which does not undergo any kind of process before it comes here, like in the case of other augmentation materials. This clot seems to be one of the best augmentation materials that fills the spaces between the surface of the screw and the bone wall, preventing its filling with connective tissues. Moreover, it actively takes part in the process of osseous proliferation by means of the blood factors it contains. Another advantage of this clot is that it can be easily removed when the time comes to make room for the newly proliferated bone on the interface. Through all these aspects, the presence of the clot greatly facilitates the process of bone proliferation on the interface. Easier new bone proliferation in the thread channels of the implant than past the threads was reported by other authors [39]. Some authors argue that this aspect is facilitated by the large number of platelets in the fibrin network, better represented in the thread channels [40,41].

Together with the coagulation processes and platelet activation, an inflammatory response is set off towards the aggression that accompanies the surgical intervention. After the platelets that come into the peri-implant space first, inflammatory cells are represented by neutrophils and monocytes. The first are the neutrophils, which reach maximum levels between 24 and 48 h, then the monocytes, which change to macrophages and reach the maximum level after 48 h [42]. The leukocytes in the peri-implant space are activated by the cytokines released by the platelets, like β-thromboglobulin (β-TG) and PDGF. The activated leucocytes release inflammation mediators like IL-1, IL-6, IL-8, tumor necrosis factor α (TNF-α), and the Macrophage Colony-Stimulating Factor (M-CSF) [42]. By the manner that the events unfold, the hematoma in the peri-implant space is similar to a fracture hematoma, expressing the same signaling molecules. The Growth Factor- β (TGF-β) is released 24 h from the lesion. Bone morphogenetic proteins (BMP) and growth and differentiating factors (GDF) are also expressed. These factors promote bone formation [43].

The complex interaction of the signaling molecules in the peri-implant space initiates and maintains the mobilization of the cells with osteogenic potential, their migration through the temporary matrix of the clot towards the surface of the implant, and their differentiation to the osteoblasts responsible for the secretion of the bone components necessary for the reparatory processes [44,45].

The cells with osteogenic potential are those recruited from the hematogenous marrow, periosteum, and endosteum, and the perivascular pericytes brought into the wound together with the angiogenesis process [46]. They migrate in the temporary matrix of the fibrin clot towards the surface of the implant. This process is mediated by the factors released by platelets and leukocytes. During this migration, they differentiate themselves from osteoblasts, which colonize the surface of the implant and start to secrete bone matrix. It was demonstrated on a pig model that the migration process of the osteoprogenitor cells towards the surface of the implant takes 24 h from the insertion of the implant [47]. The osteoblasts that reached the surface of the implant start the secretion of bone material deposited on the surface of the implant. This process is called contact osteogenesis [48]. This is how the formation of plexiform bone (immature bone tissue) is formed. This process unfolds in an appositional manner from the surface of the implant to the margins of the bone defect.

A part of the osteoblasts resulting from the differentiation of the osteoprogenitor cells does not form the fibrin–leukocytes clot reaching the surface of the implant but, instead, synthesize the bone matter that will be bedded on the surface of the bone defect. This type of bone formation is called distance osteogenesis [48]. During this process, plexiform bone is formed, which will be bedded by apposition from the wall of the bone defect to the surface of the implant. Over the two types of osteogenesis, bone is laid in the space between the surface of the implant and that of the bone wall from two different directions. By marking the bone with fluorochrome, some authors came to the conclusion that contact osteogenesis is 30% faster than distance osteogenesis [31].

The first bone matter synthetized by the osteoblasts and laid on the surface of the implant are the components of the bone matrix, which primarily contain collagen fibers incorporated into the fundamental substance. Until the onset of the mineralization process, it is called osteoid [10]. The next phase of bone formation is represented by the plexiform bone tissue, which rapidly proliferates by 30–50 μm/day. This type of bone is rich in cells and has the collagen fibers disposed of without a rigorous order. This type of tissue has a low mineral content, so its mechanical resistance is low. Most of the time, its existence is temporary, being replaced by mature lamellar bone. An exception is the bone next to the sutures of the calvaria and that from the insertion place of some tendons. As some specialists assert, early osseointegration in an experimental model is twice as fast as in humans [25,49].

The plexiform bone initially formed in the space between the surface of the implant and the surface of the bone defect will undergo the process of remodeling towards mature lamellar bone with better mechanical strength. This remodeling process is considered the late phase of peri-implant healing. The remodeling also includes the bone in the interface depth, which, at the moment of drilling, the orifice for the implant suffers microlesions 1–2 mm deep from the surface of the experimental bone defect [29]. The remodeling process also includes the host bone, which surrounds the implant up to a certain distance, to restore the total resistance of the bone weakened by the drilling. Some specialists assert that the remodeling processes unfold over a long period of time, which could exceed 6 months [30]. The remodeling of the bone on the interface takes place during the whole existence of the implant in direct relation with the biomechanical forces acting upon the bone surrounding the implant.

As a result of the peri-implant proliferated bone remodeling, two types of mature lamellar bone are formed: spongy bone and compact bone, also called cortical bone. The two bone types contain the same structural components: osseous lamellae and osseous cells, but they differ from each other in the disposition of the lamellae and the proportion between the marrow space and the osseous substance. In the spongy bone, the lamellae are adjacently sited and form splitting trabeculae, which establish large spaces filled with marrow between them. In the compact bone, the osseous lamellae are concentrically sited around a narrow channel forming Haversian systems [10].

The building of the mature lamellar bone around the implant is the desired final result. The remodeling of the bone in the peri-implant space is doubled by the remodeling of the bone in the depth of the interface, with the final result being the stabilization of the implant on its site [50].

Following up on the dynamics of the reparatory processes in the healing chamber, it is emphasized that they have a certain complexity and evolution. We noticed that the reparatory processes start during the first week after the insertion, but they do not immediately fill the healing chamber to its entire length. The fact that, after 7 days, there are osseous proliferation processes only in the third towards the endosteum of the healing chamber demonstrates that they have a starting point when they spread along the chamber. This starting point is the endosteum, which produces osteoprogenitor cells very quickly. This expansion of the newly proliferated bone towards the middle of the healing chamber is very well emphasized during day 14, when the healing chamber is filled with newly proliferated bone on two-thirds of its length, respectively, the third towards the endosteum and the middle one. Taking into account the evolution and the area occupied on day 14, we consider that the proliferated bone in the healing chamber up to this moment is totally of endosteal origin. Moreover, we noticed this in previous studies performed on rabbit cortical bone, where, 14 days after the implantation, the surface occupied by bone of endosteal–medullary origin was very large, while the proliferation with the periosteal starting point was just at the beginning [51]. In a similar study to ours, performed on rabbit cortical bone as well, Soto-Peñaloza et al. [25] noticed the presence of newly formed bone tissue with a high level of cells and vessels after 2 weeks. The difference between our study and this last one was that they did not report differences in osseous proliferation along the healing chamber.

In our study, the healing chamber was occupied by bone tissue along its whole length after 4 weeks, even if the bone was slightly different from one section to the other of the healing chamber. It is worthy of note that, at this moment of the experiment, we noticed remolding processes in the lamellar bone with the proviso that they were in an incipient stage. The presence of bone tissue in the healing chamber after 4 weeks, with incipient aspects of remolding to lamellar bone, was noticed by the above-mentioned authors as well [25].

The healing chambers are obtained by adapting the surgical technique by ensuring an adequate relation between the diameters of the osseous orifice and that of the implant, and the advantage is that these empty spaces, doubled by the presence of uncompressed fragments, favor phagocytosis activity and the new formation of bone [9]. In a study on dogs, Jimbo et al. [52] compared the osseointegration of implants inserted in a small orifice that did not ensure healing chambers with others in which they used a bigger orifice that ensured healing chambers. They noticed that the presence of healing chambers ensured a significantly higher quality of newly formed bone.

The proven utility of the healing chambers resulted from the insertion of implants in orifices with a larger diameter than the diameter of the implant body led to the idea of manufacturing implants with healing chambers already included in their structure. Such an implant with healing chambers between the threads was compared with a conventional implant in a study on rabbits. The authors came to the conclusion that the implants with already-included healing chambers significantly accelerated the osseointegration process, whether the implants underwent a surface treatment or not [15].

In another study, rabbit implants with conventional macro geometry and implants with healing chambers shaped as circular curves distributed in the body of the implant were comparatively tested. The conclusion they reached was that the implants with healing chambers presented a significant improvement in osseointegration, compared to the conventional implants [53,54]. In a study on eight Beagle dogs, they experimented with the osseointegration of 3.7 mm diameter implants in 3.0 mm, 3.25 mm, and 3.5 mm diameter orifices. This method of insertion ensured the formation of healing chambers of 30%, 50%, and 75% of the height of the thread. After 3 weeks, all of the healing chambers presented bone tissue, which supports the bone formation of the intramembranous type noticed in the healing chambers with different sizes [14]. In other studies on dogs, Bonfate et al. [55] tested implants with healing chambers with an unprocessed surface and processed with acid. After 4 weeks, they did not notice significant differences in terms of the percentage values of bone–implant contact. Another study aimed at the early osseointegration of implants with different configurations of the healing chamber by placing them in the Beagle’s mandible. The conclusion they reached was that, after 3 weeks, the healing chambers were filled with bone tissue, and after 5 weeks, they noticed initial stages of remolding to lamellar bone. The results demonstrate that more configurations of the healing chambers can contribute to the stimulation of early osseointegration of the implants [17]. Currently, there is more interest in reducing the time between implant placement and loading [56,57,58,59].

The use of implants with healing chambers in case of weak bones is arguable; that is why a surgical technique called osseodensification was conceived [60]. The technique consists of drilling without extraction with controlled plastic osseous deformation and autograft by densification. Some researchers managed with this technique to obtain healing chambers in the bone of a lesser quality without any decrease in the stability of the implant or in the success rate [16,19].

The technical progress in the last years allows for the 3D design of titanium implants with healing chambers. Most studies related to the design of the healing chambers were mainly based on the shape and pace of the thread [61] and on the diameter of the insertion orifice [62]. If the conventional implants usually present healing chambers with the same shape over the entire length of the implant of a 3D design, different chambers on the same implant can be obtained [63]. The 3D designers highly recommend the vee and eagle’s beak shapes for the healing chambers, with the proviso that they have not been tested enough in clinical practice [63].

In what concerns the depth and the height of the healing chambers, some authors suggest that they should not surpass certain limits. The risk is that an oversized chamber cannot be completely filled with blood, which would significantly compromise the osseointegration process [17,63].

We chose an implantation method to ensure a 150 µm healing chamber, the maximum permitted. We considered it to be a large size, especially when related to a small animal. Following the osseous proliferation process in the healing chambers over 28 days, we noticed that it takes certain stages, meaning a certain evolution. We did not notice a proliferation of connective tissue over that of osseous tissue on any check days (7, 14, and 28) on the entire length of the healing chamber. These aspects demonstrate that the blood totally filled the healing chamber, ensuring proper conditions for the sequential development of osteogenesis with bone formation, which filled the entire space of the chamber in the end [51,64].

The limitations of this study are given by the fact that the results obtained on the experimental animals cannot be 100% extrapolated to humans. Further investigations are necessary to determine how these findings translate to real-world clinical scenarios involving functional load. The specialists point out that there are differences even between related species, like rodents, to which the extrapolation is 70% maximum [65]. Moreover, there are differences between the bones used in the experiment and the jaw bones in which the dental implants are inserted.

## 5. Conclusions

The healing chambers ensured very proper conditions for the development of the early osseous repairing processes in case of implants inserted into the cortical bone of the rabbit. What is particular for our study is that we noticed that the repairing processes do not trigger instantly across the entire length of the healing chamber, but they have a starting point from where they spread gradually to fill the healing chamber. The starting point proved to be the endosteum, demonstrated by the fact that, on day 7, there was newly proliferated bone only on the third of the endosteum of the healing chamber. On day 14, the bone expanded to the middle third, and on day 28, it was present along the entire length of the healing chamber. At no point during this experiment did we notice the tendency of unwanted tissue proliferation, like the connective ones, which demonstrates that the 150 µm height of the healing chamber does not present a risk from this point of view for the cortical bone of the rabbit.

## Data Availability

The raw data generated throughout this study can be obtained from the first author.
